# Association of vitamin D receptor gene variants with polycystic ovary syndrome: A case control study

**Published:** 2015-12

**Authors:** Touraj Mahmoudi, Keivan Majidzadeh-A, Hamid Farahani, Mojgan Mirakhorli, Reza Dabiri, Hossein Nobakht, Asadollah Asadi

**Affiliations:** 1 *Gastroenterology and Liver Diseases Research Center, Shahid Beheshti University of Medical Sciences, Tehran, Iran.*; 2 *Cancer Genetics Department, Breast Cancer Research Center (BCRC), ACECR, Tehran, Iran.*; 3 *Department of Physiology, School of Medicine, Qom University of Medical Sciences, Qom, Iran.*; 4 *Internal Medicine Department, Semnan University of Medical Sciences, Semnan, Iran.*; 5 *Department of Biology, Faculty of Science, University of Mohaghegh Ardabili, Ardabil, Iran.*

**Keywords:** *Insulin*, *Insulin receptor*, *Polycystic ovary syndrome*, *Vitamin D receptor*

## Abstract

**Background:**

Vitamin D and insulin play an important role in susceptibility to polycystic ovary syndrome (PCOS), and therefore vitamin D receptor (*VDR*), parathyroid hormone (*PTH*), and insulin receptor (*INSR*) gene variants might be involved in the pathogenesis of PCOS.

**Objective:**

The present study was designed to investigate the possible associations between polymorphisms in *VDR*, *PTH*, and *INSR* genes and the risk of PCOS.

**Materials and Methods:**

*VDR*, *PTH*, and *INSR* gene variants were genotyped in 35 women with PCOS and 35 controls using Polymerase chain reaction – Restriction fragment length polymorphism method. Furthermore, serum levels of glucose and insulin were measured in all participants.

**Results:**

No significant differences were observed for the *VDR FokI, VDR Tru9I, VDR TaqI, PTH DraII, INSR NsiI,* and *INSR PmlI* gene polymorphisms between the women with PCOS and controls. However, after adjustment for confounding factors, the *VDR BsmI* “Bb” genotype and the *VDR ApaI* "Aa" genotype were significantly under transmitted to the patients (p= 0.016; OR= 0.250; 95% CI= 0.081-0.769, and p= 0.017; OR= 0.260; 95% CI= 0.086-0.788, respectively). Furthermore, in the women with PCOS, insulin levels were lower in the participants with the *INSR NsiI* "NN" genotype compared with those with the "Nn + nn" genotypes (P= 0.045).

**Conclusion:**

The results showed an association between the *VDR* gene *BsmI* and *ApaI* polymorphisms and PCOS risk. These data also indicated that the *INSR* "NN" genotype was a marker of decreased insulin in women with PCOS. Our findings, however, do not lend support to the hypothesis that *PTH* gene *DraII* variant plays a role in susceptibility to PCOS.

## Introduction

Polycystic ovary syndrome (PCOS), a highly complex and heterogeneous disorder with unclear etiology, is probably the most common endocrine disorder, which has a strong genetic component (1). The underlying causes of PCOS are not completely known. However, in addition to the menstrual disturbance and hyperandrogenism, PCOS patients demonstrate an increased prevalence of type 2 diabetes mellitus, impaired glucose tolerance, hyperinsulinemia, insulin resistance, and obesity (2-4). On the other hand, our previous study and other studies have demonstrated that insulin resistance and obesity have a negative effect on serum levels of 25-hydroxyvitamin D [25 (OH) D] and a positive effect on parathyroid hormone (PTH) concentrations in the women with PCOS (5-7). Furthermore, these studies have also shown higher serum levels of 25 (OH) D, phosphorous, and PTH in women with PCOS than do controls (5, 7).

The pleiotropic biological actions of 1, 25-dihydroxyvitamin D [1, 25 (OH) 2D] are mediated by the vitamin D receptor (VDR) through its ability to modulate the expression of target genes. The *VDR* gene polymorphisms have been shown to be associated with obesity, insulin resistance, serum levels of testosterone, Luteinizing hormone, PTH, and 25 (OH) D (8-12). Furthermore, in our previous study a significant association was found between *VDR* gene *ApaI* polymorphism and risk of PCOS (13). In addition, the previous studies have demonstrated significant associations between *PTH* gene polymorphisms and serum levels of PTH and calcium (14). Finally, in recent years, the associations between various *INSR* genetic variants and PCOS have been examined in several epidemiologic studies, and the results were contradictory. Some investigations have shown significant associations between *INSR* gene SNPs and the risk of PCOS (15). In contrast, other studies have found no association (16, 17).

Based on these considerations, the aim of the present study was to investigate whether the *FokI, BsmI, ApaI, TaqI,* and *Tru9I* polymorphisms of the *VDR* gene, the *DraII* polymorphism of the *PTH* gene, and the *PmlI* and *NsiI* polymorphisms of the *INSR* gene were associated with PCOS risk. Furthermore, the possible relationships of the gene variants with insulin resistance and serum levels of insulin were also evaluated. 

## Materials and methods

**Participants**

This case control study was conducted in the Gastroenterology and Liver Diseases Research Center, Shahid Beheshti University of Medical Sciences, Tehran, Iran between July 2011 and September 2012. All participants were provided informed consent to the study and the Ethics Committee of Gastroenterology and Liver Diseases Research Center reviewed and approved this study.

35 women with PCOS as the case group and 35 healthy women as controls were selected. The diagnosis of PCOS was made according to (a) the presence of menstrual dysfunction i.e. oligomenorrhea (fewer than six menstrual periods in the preceding year) or amenorrhea (absence of periods for more than 6 months), and (b) clinical hyperandrogenism (i.e. hirsutism: Ferriman-Gallwey score>6) (18) and/or hyperandrogenemia, and (c) the exclusion of other related disorders, including nonclassic congenital adrenal hyperplasia, androgen secreting tumors, Cushing's syndrome and hyperprolactinaemia. These diagnostic criteria were in accordance with the National Institute of Child Health and Human Development criteria (19). The controls had normal menstrual cycles and none of them had clinical evidence of hyperandrogenism. All 70 women were Iranian and genetically unrelated. 

**Biochemical measurements**

Blood sampling was performed between days 2 and 6 of a menstrual cycle in the control subjects or during a spontaneous bleeding episode or progestin-induced menstrual cycle in the PCOS patients. Blood samples were collected after an overnight fast and were immediately centrifuged, and sera were separated and frozen at –20^o^C until assayed. In all of the 70 participants serum concentrations of glucose and insulin were measured. 

The homeostasis model assessment of insulin resistance index (HoMA-IR) was calculated according to the formula: [fasting insulin (µIU/ml) × fasting glucose (mg/dl)] /405 (20). The serum concentration of insulin was determined by radioimmunoassay (RIA) method (DRG instruments GmbH, Marburg, Germany). Serum glucose measurements were performed using standard methods (CinnaGen Inc., Tehran, Iran). The intra- and inter assay coefficients of variation were 2.2 and 6.5% for insulin, respectively.

**Genotype analysis**

Blood samples for molecular genetic studies were collected in tubes containing EDTA as an anticoagulant and store at 4C. Genomic DNA was purified from whole blood using a commercial isolation kit (BioNEER, Daejeon, Korea) according to manufacturer’s instructions and was amplified by PCR. Genotyping was carried out by RFLP. Details of the studied SNPs, PCR Primers, PCR condition, RFLP condition, the length of PCR products and RFLP products are shown in Table I.

In this study, the SNPs studied were chosen based on their common use in previous genetic epidemiology studies and degree of heterozygosity.

The PCR products were digested overnight by corresponding restriction enzymes (Fermentas, Leon-Rot, Germany) and the RFLP products were run on 2 to 3% agarose gels, and stained with ethidium bromide for visualization under UV light. Genotypes were expressed in RFLP nomenclature: upper case letters denote the absence of a restriction site, while lower case letters indicated the presence of a restriction site. In other words, *VDR, PTH*, and *INSR* genotypes of each participants were identified according to the digestion pattern and alleles according to the presence ("f" or "b" or "a" or "r" or "t" or "d" or "n" or "p") or absence ("F" or "B" or "A" or "R" or "T" or "D" or "N" or "P") of the *FokI, BsmI, ApaI, Tru9I, TaqI, DraII, NsiI*, and *PmlI* sites, respectively. The "F", "B", "A", "R", "T", "D", "N", and "P" alleles correspond to "C", "A", "A", "G", "T", "A", "G", and "T" nucleotides, respectively. Approximately 10% of the samples were randomly selected and genotyped in duplicate for confirmation and the results were 100% concordant.

**Table I:** Information for the studied markers in the *VDR, PTH*, and *INSR* genes

**Gene** **(SNP)**	**SNP reference number**	**Location** **(Base change)**	**Forward primer** **Reverse primer**	**PCR** **condition**	**PCR** **fragment** **size (bp)**	**Restriction** **enzyme,** **Incubation temperature**	**RFLP** **fragments size (bp)**
VDR (FokI)	rs10735810	Exon 2 (C/T)	5'-AGCTGGCCCTGGCACTGACTCTGCTCT-3' 5'-ATGGAAACACCTTGCTTCTTCTCCCTC-3'	35 cycles 93C 45s, 66C 30s, 72C 45s	265	FoKI, 55C	169, 96
VDR (BsmI)	rs1544410	Intron 8 (A/G)	5’-GGCAACCTGAAGGGAGACGTA-3’ 5’-CTCTTTGGACCTCATCACCGAC-3’	35 cycles 93C 45s, 66C 30s, 72C 45s	461	BsmI, 37C	258, 203
VDR (ApaI)	rs7975232	Intron 8 (A/C)	5’-CAGAGCATGGACAGGGAGCAAG-3’ 5’-GCAACTCCTCATGGCTGAGGTCTCA-3’	35 cycles 93C 45s, 66C 30s, 72C 45s	740	ApaI, 37C	530, 210
VDR (Tru9I)	rs757343	Intron 8 (A/G)	5’-AATACTCAGGCTCTGCTCTT-3’ 5’-CATCTCCATTCCTTGAGCCT-3’	35 cycles 93C 45s, 56C 30s, 72C 45s	331	Tru9I, 65C	178, 153
VDR (TaqI)	rs731236	Exon 9 (C/T)	5’-CAGAGCATGGACAGGGAGCAAG-3’ 5’- GCAACTCCTCATGGCTGAGGTCTCA-3’	35 cycles 93C 45s, 66C 30s, 72C 45s	740	TaqI, 65C	495,290, 245, 205
PTH (DraII)	rs6256	Exon 3 (A/C)	5’-CATTCTGTGTACTATAGTTTG-3’ 5’-GAGCTTTGAATTAGCAGCATG-3’	35 cycles 93C 45s, 56C 30s, 72C 45s	600	DraII, 37C	420, 180
INSR (NsiI)	rs2059806	Exon 8 (A/G)	5’-CGGTCTTGTAAGGGTAACTG-3’ 5’-GAATTCACATTCCCAAGACA-3’	35 cycles 93C 45s, 56C 30s, 72C 45s	324	NsiI, 37C	239, 85
INSR (PmlI)	rs1799817	Exon 17 (C/T)	5’-CCAAGGATGCTGTGTAGATAAG-3’ 5’-TCAGGAAAGCCAGCCCATGTC-3’	35 cycles 93C 45s, 56C 30s, 72C 45s	317	PmlI, 37C	274, 43

*VDR:* Vitamin D receptor*; PTH:* Parathyroid hormone*; INSR: *Insulin receptor.

**Statistical analysis**

To examine the normality of distribution of continuous variables between the studied women in two groups, the Kolmogorov-Smirnov goodness-of-fit test was used and where necessary, log transformation was performed. Because the values of HoMA- IR and serum insulin and glucose were skewed, they were log transformed for the analysis, and their geometric mean (geometric standard error of the mean) was presented.

Comparisons of the distribution of the allele and genotype frequencies were performed using the Chi-square test (χ2). The Chi-square test was also used to examine whether genotype frequencies for each polymorphism satisfied the Hardy-Weinberg equilibrium. Differences between genotype groups in biochemical parameters were assessed by ANOVA or analysis of covariance when appropriate. The odds ratios (OR) given with the respective 95% confidence intervals (95% CI) were estimated in the alleles and genotypes with a statistically significant difference among study groups. Data were analyzed using Statistical Package for the Social Sciences, version 15.0, SPSS Inc, Chicago, Illinois, USA, and the difference was considered significant at P< 0.05.

## Results

Table II presents the selected characteristics of cases (age range, 19-42 years) and controls (age range, 19-44 years). There were no significant differences between case and control groups in age, Body mass index (BMI), insulin resistance, and serum level of insulin. However, the cases had significantly lower serum levels of glucose compared with the controls (p= 0.023) and the effect of PCOS on the variable remained significant after adjustment for age and BMI (p= 0.019).

Table III shows the genotype and allele distributions for the *FokI, BsmI, ApaI, Tru9I*, and *TaqI* polymorphisms of the *VDR* gene, the *DraII* polymorphism of the *PTH* gene, and the *NsiI* and *PmlI* polymorphisms of the *INSR* gene in women with PCOS and controls. None of the genotype frequency distributions deviated significantly from the Hardy-Weinberg equilibrium (Table III). No significant differences were observed for the *FokI, Tru9I, TaqI, DraII, NsiI,* and *PmlI* polymorphisms in either genotype or allele frequencies between the cases with PCOS and controls. However, analysis of the BsmI and ApaI polymorphisms revealed significant differences between women with PCOS and the control women (Table III). 

After adjustment for age and BMI, the *VDR* “Bb” genotype and "Aa" genotype were significantly under transmitted to the patients (p= 0.016; OR=0. 250; 95% CI= 0.081-0.769, and p= 0.017; OR= 0.260; 95% CI= 0.086-0.788, respectively).

After adjustment for age and BMI in the controls, women carrying the *VDR* "AA" or "BB" genotype had lower serum glucose levels as compared with individuals in the "Aa + aa" (n= 8, n= 27, respectively, 120.64 ± 1.09 versus 91.74 ± 1.04, P= 0.007) or "Bb+bb" (n= 5, n= 30, respectively; 119.58 ± 1.10 versus 94.43 ± 1.05, P= 0.037) genotypes. Furthermore, in the controls, serum level of insulin and insulin resistance in the women with the *INSR* "pp" genotype compared with those with the "PP + Pp" genotypes were lower and the difference remained significant after adjustment for age and BMI (n= 18, n= 17, respectively; insulin 9.28 ± 1.33 versus 21.05 ± 1.23, p= 0.036; HoMA- IR 2.15 ± 1.35 versus 5.30 ± 1.26, p= 0.041). In addition, in the women with PCOS, insulin levels was lower in the women with the *INSR NsiI* "NN" genotype compared with those with the "Nn + nn" genotypes and the difference remained significant after adjustment for age and BMI (n= 18, n= 17, respectively; 13.25 ± 1.13 versus 18.61 ± 1.14, p= 0.045).

**Table II:** Clinical and biochemical variables of the studied groups

	**Control group (n=35)**	**Case group (n=35)**	**P**_1_ b	**P**_2_ c
Age (years)	30.29 (1.17)	28.43 (0.80)	0.196	-
BMI (kg/m^2^)	26.30 (0.63)	26.98 (0.83)	0.513	-
HoMA –IR^d^	3.33 (1.22)	3.33 (1.11)	0.999	0.848
Serum glucose (mg/dl)^d^	97.67 (1.04)	86.35 (1.03)	0.023	0.019
Serum insulin^e ^(µIU/ml)^d^	13.81 (1.21)	15.63 (1.10)	0.558	0.687

a Data are presented as mean ± standared error (SE). Geometric mean (geometric standard error of the mean) is presented for HoMA– IR and serum glucose and insulin,

b P_1_ is the unadjusted p values (One-way ANOVA test),

c P_2_ is the adjusted p-values for age and body mass index (ANCOVA test),

d Significance was tested on log-transformed values.

**Table III:** Association between genotypes and alleles of *VDR, PTH,* and *INSR* gene polymorphisms and risk of PCOS

**Gene/SNP **	**Control group (n=35)** a	**Case group ** **(n=35)** a	**P-value**
Genotype and allele-wise comparisons			
*VDR/FokI*			
FF: Ff: ff	24 (68.6): 10 (28.6): 1 (2.8)	16 (45.7): 17 (48.6): 2 (5.7)	0.153
F: f	58 (82.9): 12 (17.1)	49 (70.0): 30 (21.0)	0.073
*VDR/BsmI* b			
BB: Bb: bb	5 (14.3): 23 (65.7): 7 (20.0)	10 (28.6): 12 (34.3): 13 (37.1)	0.031
B: b	33 (47.1): 37 (52.9)	32 (45.7): 38 (54.3)	0.865
*VDR/ApaI* c			
AA: Aa: aa	8 (22.9): 21 (60.0): 6 (17.1)	15 (42.9): 11 (31.4) : 9 (25.7)	0.054
A: a	37 (52.9): 33 (47.1)	41 (58.6): 29 (41.4)	0.496
*VDR/Tru9I*			
RR: Rr: rr	27 (77.1): 8 (22.9): 0 (0.0)	28 (80.0): 6 (17.1): 1(2.9)	0.521
R: r	62 (88.6): 8 (11.4)	62 (88.6): 8 (11.4)	1.000
*VDR/ TaqI*			
TT: Tt: tt	15 (42.9): 16 (45.7): 4 (11.1)	15 (42.9): 14 (40.0): 6 (17.1)	0.766
T: t	46 (65.7): 24 (34.3)	44 (62.9): 26 (37.1)	0.724
*PTH/DraII*			
DD: Dd: dd	0 (0.0): 16 (45.7) : 19 (54.3)	1 (2.9): 9 (25.7): 25 (71.4)	0.151
D: d	19 (27.1): 51 (72.9)	11 (15.7): 59(84.3)	0.099
*INSR/NsiI*			
NN: Nn: nn	21 (60.0) : 12 (34.3) : 2 (5.7)	18 (51.4): 12 (34.3): 5 (14.3)	0.468
N: n	54 (77.1): 16 (22.9)	48 (68.6): 22 (31.4)	0.254
*INSR/PmlI*			
PP: Pp: pp	1 (2.9) : 16 (45.7) : 18 (51.4)	0 (0.0): 14 (40.0): 21 (60.0)	0.506
P: p	18 (25.7): 52 (74.3)	14 (20.0): 56 (80.0)	0.424

*VDR:* Vitamin D receptor*; PTH:* Parathyroid hormone*; INSR: *Insulin receptor.

a Data are presented as number (%),

b chi-square test for genotype-wise comparisons: BB/Bb + bb (p= 0.152; OR= 2.400, 95% CI= 0.725-7.949), Bb/BB + bb (p= 0.010; OR= 0.272, 95%CI= 0.101-730), and bb/ BB + Bb (p= 0.117; OR= 2.364, 95%CI= 0.807-6.927) test models, respectively.

c chi-square test for genotype-wise comparisons: AA/Aa+aa (p=0.079; OR=2.531, 95% CI=0.899-7.124), Aa/AA+aa (p=0.018; OR=0.306, 95%CI=0.114-0.817), and aa/AA+Aa (p= 0.079; OR= 1.673, 95%CI= 0.524-5.341) test models, respectively.

## Discussion

Based on our results, significant associations between *VDR* gene *BsmI* and *ApaI* polymorphisms and risk of PCOS were found. The *BsmI* "Bb" or ApaI "Aa" genotypes appeared to be a marker of decreased PCOS susceptibility.

Furthermore, in the women with PCOS the *INSR NsiI* "NN" genotype appeared to confer an increased risk for serum insulin level, which our knowledge has not been reported previously. 

Also, the *INSR PmlI* "pp" genotype appeared to confer a decreased risk for serum insulin levels and insulin resistance in the controls. In addition, the *VDR BsmI* "BB" or *ApaI* "AA" genotypes appeared to confer a decreased risk for serum glucose levels in the control women. 

In recent years, genes involved in insulin signaling pathway have been suggested as candidate genes for PCOS. Our finding is in line with the other studies showing no significant association between the *INSR* gene polymorphisms and the risk of PCOS (16, 17).

However, positive associations also have been reported (15). Inconsistent results may be due to false positive results, population differences in allele frequencies, differences in disease definition, genotyped markers, statistical methods, and the variation in environmental, particularly nutritional factors. Furthermore, the *NsiI* and *PmlI* RFLPs at exon8 and exon 17 are "synonymous" polymorphisms meaning that they do not alter the amino acid sequence of the *INSR* gene and may only be associated with risk of PCOS in some populations through linkage disequilibrium with another functional variant. In the present study, a significant association between *INSR* gene *NsiI* polymorphism and serum levels of insulin was also observed that is in agreement with the previous reports (21); to our knowledge, ours is the first study that demonstrates the association in women with PCOS. Furthermore, the results are in line with the previous studies (22, 23) showing significant associations between the *INSR* *PmlI* polymorphism and serum levels of insulin and insulin resistance. However, the molecular mechanisms behind the genotype/phenotype associations were observed for the *INSR* gene polymorphisms remain unexplained. 

In this study, significant associations between *VDR BsmI* and *ApaI* gene polymorphisms and risk of PCOS were observed that are in agreement with our previous report (13), to our knowledge, the present study is the first to show that *VDR* gene *BsmI* polymorphism is associated with risk of PCOS. In our previous study, the *ApaI* “Aa” genotype was a marker of decreased PCOS susceptibility, whereas “aa” genotype was associated with an increased risk for PCOS (13). Furthermore, in the present study, significant associations between these two SNPs and serum levels of glucose in the controls were observed that they were in agreement with the previous reports (24). The *BsmI* and *ApaI* polymorphisms are located in intron 8 at the 3' end of the *VDR* gene. The 3' untranslated region of genes is known to be involved in the regulation of gene expression, especially through regulation of mRNA stability. Some studies have suggested that, the *BsmI* and *ApaI* polymorphisms are associated with differences in *VDR* mRNA expression level and stability (25). Furthermore, Whitfield *et al* (26) have shown that the “b” allele of *BsmI* polymorphism appears to be more active than the “B” allele. On the other hand, in accordance with this study it has been reported that fasting plasma glucose and prevalence of glucose intolerance were significantly lower in individuals with *ApaI* "AA" genotype compared with those with "aa" genotype, (24) and the effect of *VDR* *ApaI* polymorphism on insulin secretion has been reported (27). In addition, our previous study showed that the "aa" genotype and the "bb" genotype conferred a decreased risk of obesity in women with PCOS (13). Finally, insulin secretion is a calcium-dependent process and 1, 25 (OH) 2 D-VDR complex stimulates the expression of insulin receptor gene (28). 

Accordingly, it is possible that *VDR* gene polymorphisms through affecting the insulin signaling pathway play a role in pathogenesis of PCOS. However, because these polymorphisms are largely nonfunctional, it seems that linkage disequilibrium with another unknown functional variant of the *VDR* gene is assumed to explain the associations observed. 

Furthermore, it is possible that *VDR* gene polymorphisms through affecting PTH- vitamin D axis play a role in pathogenesis of PCOS. Consistent with the view, it has been shown the associations between the *VDR* gene polymorphisms and serum levels of PTH (11) and 25 (OH) D (12), and vitamin D-VDR complex inhibit the secretion and synthesis of PTH (29). 

The present study has several limitations. A limitation of the present study is its sample size. Considering the small sample size, the genotype differences may be attributable strictly to chance. Another potential limitation is our lack of information on serum levels of vitamin D and PTH, which could modify the effects observed here. Accordingly, it could not completely rule out the possibility of chance findings. Nevertheless, the possibility of true finding should not be excluded.

In conclusion, *VDR* gene variants appeared to be markers of decreased PCOS susceptibility. Our findings also suggested that INSR gene variant could affect serum level of insulin in women with PCOS. However, further studies are needed to confirm the findings and clarify the biological mechanisms by which these polymorphisms influence the PCOS risk.
